# ZIP4 Is a Novel Cancer Stem Cell Marker in High-Grade Serous Ovarian Cancer

**DOI:** 10.3390/cancers12123692

**Published:** 2020-12-09

**Authors:** Qipeng Fan, Wen Zhang, Robert E. Emerson, Yan Xu

**Affiliations:** 1 Department of Obstetrics and Gynecology, Indiana University School of Medicine, 950 W. Walnut St. R2-E380, Indianapolis, IN 46202, USA; qifan@iu.edu (Q.F.); zhangwencyyy@163.com (W.Z.); reemerso@iupui.edu (R.E.E.); 2 Pharmaceutical Research Center, Beijing Chao-Yang Hospital, Capital Medical University, No.8 Gongti Nanlu, Beijing 100020, China

**Keywords:** ALDH, cancer stem cell (CSC), high-grade serous ovarian cancer (HGSOC), NOTCH3, ZIP4

## Abstract

**Simple Summary:**

Ovarian cancer is the most deadly gynecologic cancer. The treatment options for ovarian cancer, and for the recurrent cancer in particular, are limited. One of the major obstacles is the presence of drug-resistant cancer stem cells. The aim of this study is to identify and characterize a new cancer stem marker, namely ZIP4. ZIP4 is a transporter for human essential element zinc. Our results have shown that ZIP4 is not only a novel and potent stem cell marker, but also a target for developing innovative treatment for ovarian cancer. In addition, ZIP4 is interacting with another oncogene, NOTCH3. Both ZIP4 and NOTCH3 play important roles in tumor development in ovarian cancer. This interaction may represent a useful target for ovarian cancer treatment.

**Abstract:**

High-grade serous ovarian cancer (HGSOC) is one of the most deadly and heterogenic cancers. We have recently shown that ZIP4 (gene name *SLC39A4*), a zinc transporter, is functionally involved in cancer stem cell (CSC)-related cellular activities in HGSOC. Here, we identified ZIP4 as a novel CSC marker in HGSOC. Fluorescence-activated cell sorter (FACS)-sorted ZIP4^+^, but not ZIP4^−^ cells, formed spheroids and displayed self-renewing and differentiation abilities. Over-expression of ZIP4 conferred drug resistance properties in vitro. ZIP4^+^, but not ZIP4^−^ cells, formed tumors/ascites in vivo. We conducted limiting dilution experiments and showed that 100–200 ZIP4^+^ cells from both PE04 and PEA2 cells formed larger tumors than those from 100–200 ALDH^+^ cells in mice. Mechanistically, we found that ZIP4 was an upstream regulator of another CSC-marker, NOTCH3, in HGSOC cells. NOTCH3 was functionally involved in spheroid formation in vitro and tumorigenesis in vivo in HGSOC. Genetic compensation studies showed that NOTCH3, but not NOTCH1, was a critical downstream mediator of ZIP4. Furthermore, NOTCH3, but not NOTCH1, physically bound to ZIP4. Collectively, our data suggest that ZIP4 is a novel CSC marker and the new ZIP4-NOTCH3 axis represents important therapeutic targets in HGSOC.

## 1. Introduction

Epithelial ovarian cancer (EOC), and high grade serous ovarian cancer (HGSOC) in particular [1], is the most lethal gynecologic malignancy in the United States and worldwide, primarily due to a lack of reliable early diagnostics, high incidence of chemoresistant recurrent disease, and profuse tumor heterogeneity. One emerging model for the development of drug-resistant tumors invokes a pool of self-renewing malignant progenitors known as cancer stem cells (CSCs) or tumor initiating cells (TICs).

CSCs are a small proportion of tumor cells with tumor forming capacity, which have been identified in both blood and solid tumors, including EOC [2,3,4]. CSCs contribute directly to drug-resistance and hence represent important targets for novel therapeutic strategies aimed at eradicating ovarian cancer. HGSOC is one of the most heterogenic cancers among solid cancers [5]. Similarly, its CSC markers are also highly heterogeneous, representing one of the major challenges in targeting CSCs in EOC. More than 10 CSC markers, including side population (SP), CD133, ALDH1/2, LY6A, LGR5, EpCAM, CD133, CD44, CD34, CD24, CD117, MyD88, and CDH1 have been identified in EOC [6,7]. In addition, the Wnt, SONIC Hedgehog (SHH), NOTCH, PI3K/PTEN, MAPK, and NF-κB signaling pathways have been implicated in EOC CSC [6]. Nevertheless, neither a single CSC marker nor a combination of 2–3 markers can represent all of the highly heterogeneous EOC cell lines and/or patient-derived xenografts (PDXs). Moreover, which EOC CSC markers are functionally involved and have the most significant targeting values need to be further determined and characterized [2,6].

ZIP4 (gene name *SLC39A4*) is a zinc transporter. Although more than 20 zinc transporters exist, ZIP4 stands out as a prominent cancer-related gene [8]. Its tumor promoting roles have been reported in several cancers, including pancreatic cancer, hepatocellular carcinomas, nasopharyngeal carcinoma, breast cancer, and glioma [9,10,11,12,13]. Through genetic, cell biological, and biochemical analyses, we have recently identified several previously unknown ZIP4-related regulatory and functional processes. Specifically, we have found that ZIP4 is upregulated by a lipid growth factor, lysophosphatidic acid (LPA) mainly via PPARγ, and to a much lesser extent, via LPA’s G protein coupled receptors in EOC cells. Using the Cas9 nuclease to facilitate RNA-guided site-specific DNA cleavage (CRISPR)-mediated knockout (KO) cell lines for ZIP4 and PPARγ, we have shown that ZIP4 is functionally involved in CSC-related cellular activities including anoikis-resistance, colony-formation, spheroid-formation, drug-resistance, and LPA-induced EOC side population (SP) in HGSOC cells [14]. Intriguingly, we have shown that ZIP4 is an upstream regulator of several known CSC markers: ALDH1, OCT4, and SOX9 [14,15,16]. Moreover, the TCGA and Oncomine data suggest that the ZIP4 gene is overexpressed in EOC [5], which has been confirmed in one of our recent studies [14]. We have shown that the ZIP4 protein is overexpressed in EOC vs. benign and normal ovarian tissues (Supplementary data in Ref. [14]). Of HGSOC samples 75% expressed high levels of ZIP4, with the remaining 25% (*N* = 16) of HGSOC tissues also expressed ZIP4, albeit with lower levels. Only 1 of 4 (25%) low grade serous ovarian cancer tissue samples expressed high levels of ZIP4 and none of the other groups of tissues (ovarian endometrioid carcinoma, serous borderline ovarian cancer, and control tissues) expressed high levels of ZIP4. These data have justified the clinical relevance for ZIP4 studies in EOC. However, ZIP4 as a CSC marker has not been reported in any cancer. The only previous suggestion linking ZIP4 to its potential role in stemness in lung cancer was based on correlative and indirect data [17].

The NOTCH signaling pathway is a highly conserved cell signaling system believed to be present in all multicellular organisms. Mammals possess four different NOTCH receptors, NOTCH1-4 [18], which are single-transmembrane receptor proteins. NOTCH3 regulates CSC activities including cell proliferation, anoikis-resistance, colony-formation, drug-resistance, and SP in various cancer cells, including EOC [19,20]. TCGA analyses of 489 HGSOC tumors have revealed NOTCH activation/alteration is one of the four critical pathways altered in HGSOC [5]. The NOTCH3 gene is amplified in 20% of HGSOC and is required for proliferation and survival of these tumors [21]. Jagged-1 (Jag1) has been identified as the primary NOTCH3 ligand in ovarian carcinoma and Jag1/NOTCH3 interaction constitutes a juxtacrine loop promoting proliferation and dissemination of EOC cells within the intraperitoneal cavity [22,23]. In addition, NOTCH3 overexpression is related to the recurrence of ovarian cancer, poor prognosis, and resistance to carboplatin [24,25,26]. NOTCH3 targeting is now considered to be a novel weapon against EOC CSC [27]. Interestingly, we found that a subset of development/differentiation and/or stem cell related genes, including NOTCH, were co-upregulated in the more aggressive vs. less aggressive mouse EOC cell pairs [15]. However, how NOTCH3 is regulated in the CSC context in EOC is essentially unknown.

In this manuscript, we provide the first evidence showing that ZIP4 is a potent CSC marker for tumor formation in HGSOC using fluorescence-activated cell sorter (FACS) isolated ZIP4^+^ vs. ZIP4^−^ HGSOC cells. We analyzed CSC-like activities by measuring drug resistance, spheroid- and colony-formation, cell differentiation, and self-renew assays in vitro. We also compared the tumor initiating activity of ZIP4^+^ cells with ZIP4^−^, ALDH^+^, and ALDH^−^ cells in vivo to determine their relative potency and selectivity in tumor formation. For mechanistic studies, we focused on a novel ZIP4-NOTCH3 axis using genetic and biochemical approaches. Collectively, our data have shown that ZIP4 is not only a new CSC marker in HGSOC, but also a powerful target, due to its up-stream driver functions in regulating several other CSC markers, its functional involvement in drug-resistance and spheroid formation, and its potent tumor forming capacity. Our data also provide a basis to target the novel ZIP4-NOTCH3 axis in HGSOC.

## 2. Results

### 2.1. Differentiation in ZIP4 Expression and Isolation of ZIP4^+^ and ZIP4^−^ Cells

**Figure 1:**
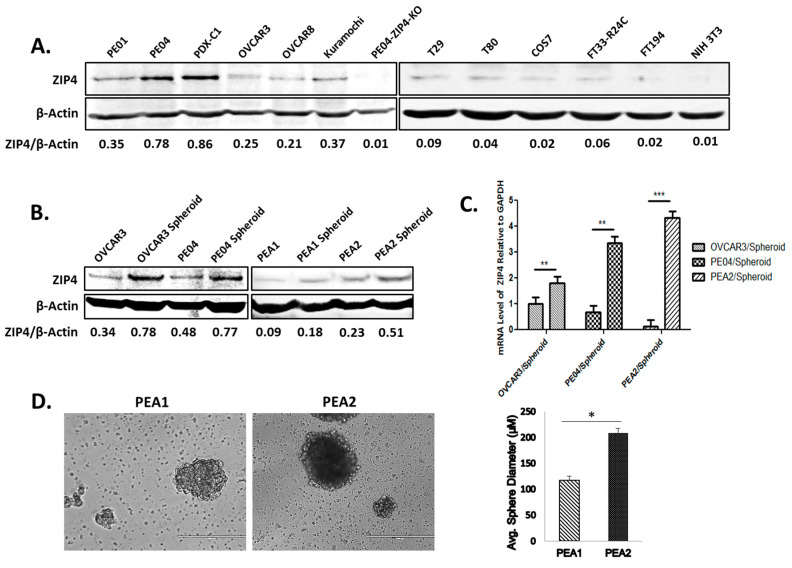
Increased protein and mRNA expression of ZIP4 in high grade serous ovarian cancer (HGSOC) cells, spheroids vs. non-malignant cells and ZIP4^+^ cell isolation. (**A**) Left panel: Western blot analysis of ZIP4 expression in HGSOC cell lines PE01, PE04, a HGSOC PDX cell line PDX-C1, OVCAR3, OVCAR8, Kuramochi, and PE04-ZIP4-KO (this ZIP4-knockout cell line is described in our previous publication [14]). The relative expression levels were quantified using the ratios of ZIP4/β-actin. Right panel: Western blot analysis of ZIP4 expression in non-tumorigenic cell lines T29, T80, COS7, FT33-R24C, FT194, and NIH3T3. (**B**) In OVCAR3, PE04, PEA1, and PEA2, ZIP4 expressed at higher levels in spheroid cells (3D) than in non-spheroid cells (2D). Same numbers of cells (5000/well into 6-well plates) were initially seeded for 3D (Costar^®^ 6-well Ultra-Low Attachment Plates) and 2D cultures. We collected cells after 3 and 14 days for the 2D and 3D culture, respectively. The ratios of ZIP4/β-actin were used for the quantitative comparisons. The uncropped Western blot images for (A) and (B) are shown in the Figure S2. (**C**) Human ZIP4 mRNA expression in HGSOC cell lines cultured in regular medium with FBS vs. 3D conditions to form spheroids. In OVCAR3, PE04, and PEA2, ZIP4 expressed at higher levels in spheroid cells (3D) than in non-spheroid cells (2D) (scale bar = 400 μm). The results were from three independent experiments. ** *p* < 0.01, *** *p* < 0.001. (**D**) Spheroids that developed from PEA2 cells are larger than those from PEA1 cells. The bars in the graph depict the average ± SD of spheroids formed in three independent experiments. * *p* < 0.05.

We have shown that ZIP4 is overexpressed in HGSOC human tissues when compared to benign and normal ovarian tissues [14]. ZIP4 was expressed in all HGSOC cell lines examined, including PE01, PE04, PEA1, PEA2, OVCAR3, OVCAR8, and Kuramochi [28,29,30,31] (Figure 1A, the left panel, and Figure 1B). We successfully generated a PDX following published methods [32,33] using ascites tumor cells from HGSOC (Stage III) patients in NOD-scid IL2Rgammanull (NSG) mice. The cell line PDX-C1, derived from this PDX, also expressed ZIP4 (Figure 1A, the left panel). The PDX tumors formed in vivo are shown in Figure S1. In contrast, several non-tumorigenic and/or immortalized cell lines, including NIH3T3, Cos7, human ovarian surface epithelial (HOSE) cell lines, T29, T80, and a human fallopian tube cell line FT194 expressed low or undetectable ZIP4 (Figure 1A, the right panel).

PE01/ PE04 and PEA1/PEA2 cell line pairs were derived from the same HGSOC patients before (PE01 and PEA1) and after (PE04 and PEA2) the onset of multidrug resistance [31,34,35]. ZIP4 expressed at higher levels in drug-resistant PE04 and PEA2 than in their drug-sensitive counterparts PE01 and PEA1 cells, respectively [14] (Figure 1A,B). In addition, ZIP4 (protein and mRNA) expressed at higher levels in spheroids, a source with enriched CSCs, than in the cells cultured in 2D tissue culture dishes (Figure 1B,C). Moreover, similar to PE04 vs. PE01 as we have shown previously [14], PEA2 cells formed larger and/or more spheroids compared to PEA1 cells (Figure 1D). These data support the CSC-related properties of ZIP4 that we have recently reported [14].

### 2.2. Isolation of ZIP4^+^ and ZIP4^−^ Cells

**Figure 2:**
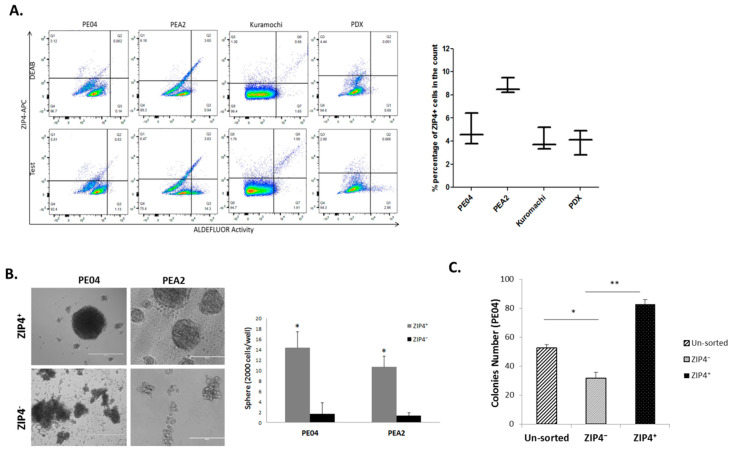
Isolation of ZIP4^+^ cells and their CSC-like activities. (**A**) ZIP4 and ALDEFLUOR-based fluorescence-activated cell sorter (FACS) analyses of HGSOC cells. The average ± SD percentage of ZIP4^+^ cells detected in each cell line and the PDX from three individual assays are shown in the right panel. (**B**) Representative tumor sphere formation assay images showing significantly enhanced sphere-forming potential in the ZIP4^+^ cells compared to the ZIP4^−^ group (scale bar = 400 μm). The bar graph depicts the average number ± SD, of spheroids formed from ZIP4^+^ cells and ZIP4^−^ cells in PEA2 and PEA1 in three independent experiments. (**C**) ZIP4^+^ cells formed significantly more colonies than their parental or ZIP4^−^ counterparts in PE04 cells. * *p* < 0.05 and ** *p* < 0.01, using Student’s *t* test.

ZIP4 has not been identified previously as a CSC marker for any cancer. To test the potential marker value of ZIP4, we identified a ZIP4 antibody suitable for FACS analysis (AF7315, R&D Systems 1:50). We established the FACS method to detect ZIP4^+^ cells in HGSOC cell lines PE04, PEA2, and Kuramochi cell lines, and the PDX cells. These cells contained 2.8–10.0% ZIP4^+^ cells (Figure 2A). As a comparison, ALDH activity sorting was also included, since it is widely accepted as an EOC CSC marker [7,36] (Figure 2A). These cells contained 1–6% ALDH^+^ cells, consistent with a previous publication [37]. ZIP4^+^, ALDH^+^ cells, and their corresponding negative cell populations were separated by FACS sorting (Figure 2A) and used for in vitro and in vivo studies. ZIP4^+^ cells displayed CSC-like activity depicted by increased spheroid- and colony-formation capacities, when compared to the unsorted parental PE04 cells and/or ZIP4^−^ cell population (Figure 2B,C).

### 2.3. Differentiation and Renewal of ZIP4, and Drug Resistance Conferred by ZIP4

**Figure 3:**
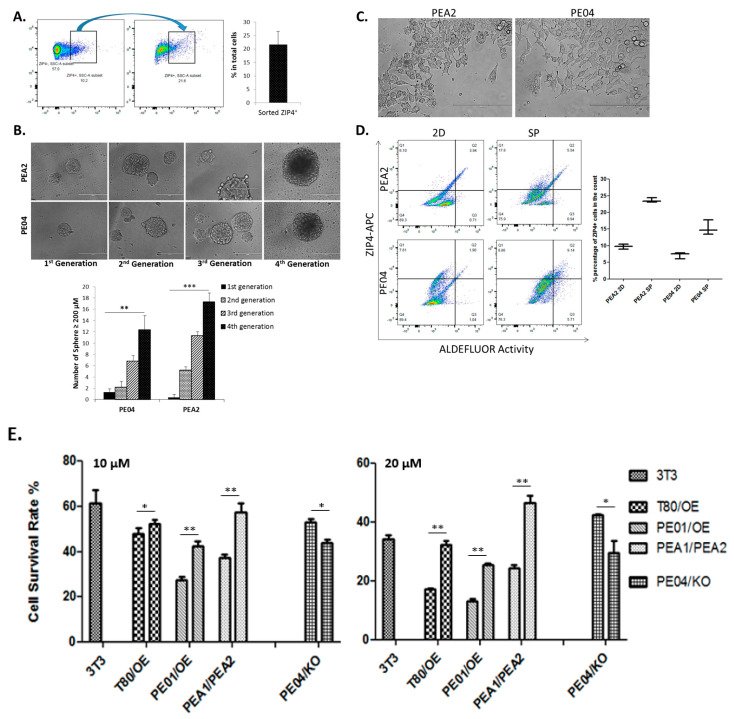
ZIP4^+^ cells self-renewal capacity and ZIP4 expression correlated with acquired resistance to cisplatin. (**A**) A representative example of flow cytometry and gating for sorted ZIP4^+^ cells (100% pure) self-renewed to ZIP4^+^ cells under differentiation conditions. The bar graph on the right depicts the self-renewing capacity of ZIP4^+^ cells from three independent studies. (**B**) Spheroids formed in each passage. The bar graph on the right depicts the sizes of the spheroids formed in three independent studies (scale bar = 400 μm). (**C**) The morphology of the differentiated cells from dissociated cells from the fourth generation spheroids (scale bar = 400 μm). (**D**) FACS analyses of ZIP4^+^ and ALDH^+^ cells in 2D differentiated cells vs. the spheroids (four time passaged) in PEA2 and PE04 cell lines. (**E**) The sensitivity to cisplatin of PE01/ZIP4-OE, PE04/ZIP4-KO, T80/ZIP4-OE, and PEA1/PEA2 cells was detected by the MTT assay. * *p* < 0.05, ** *p* < 0.01, *** *p* < 0.001.

We tested whether ZIP4^+^ cells could be differentiated into ZIP4^+^ and ZIP4^−^ cells when cultured under differentiation conditions. As shown in Figure 3A, ZIP4^+^ cells FACS sorted (100% pure) were cultured in a differentiation medium (DMEM medium with 10% FBS) for 48 h. The cells were resorted by FACS. ZIP4^+^ cells were 23.6% ± 2.1%.

In addition, we conducted spheroid renewal/differentiation experiments, similar to that described by Zhang et al. [38]. When spheroids formed from PE04 and PEA2 cells were passaged several times (initial spheroids formed under stem cell conditions were trypsinized to single cells and then rereplaced under stem cell conditions), they formed fewer but larger spheroids (Figure 3B), indicating that they had a renewal ability as described previously [38]. When the trypsinized cells were placed under differentiation conditions, they spread out as differentiated cells (Figure 3C). FACS analyses were conducted to compare ZIP4^+^ CSCs in the bulk PE04/PEA2 cells or 2D cultured differentiated cells to passaged spheroid cells. The percentages of ZIP4^+^ cells in 2D cultured differentiated cells were not significantly different from those of bulk PE04/PEA2 cells. However, the ZIP4^+^ cells were enriched from 10 to 24% and from 8 to 18% in spheroids from PEA2 and PE04 cell lines, respectively (Figure 3D). ALDH^+^ cells were also enriched in spheroid cells (Figure 3D; the negative controls in the presence of DEAB were ≤1.5%; data not shown).

CSCs are, in general, associated with multidrug-resistance [39]. In fact, high levels of efflux of the Hoechst33342 dye by ABC transporters reflect multidrug resistance, which can be detected by FACS analyses as a side-population (SP) and is a standard assay for CSCs [7]. CSCs are major obstacles for successful cancer treatment. We tested whether ZIP4 expression affected drug resistance in pairs of ZIP4 differentially expressed cell lines: T80-vector control vs. T80-ZIP4-OE cells; PE04-vector control and PE04-ZIP4-KO cells; and, PE01-vector control and PE01-ZIP4-OE cells (some of the results are published [14]). ZIP4 overexpression (OE) in cells conferred more resistance to CDDP and conversely, ZIP4-KO made the cells more sensitive to CDDP (10 and 20 μM; the results after 48 h are shown in Figure 3E; the results after 72 h had the same trend; not shown), suggesting that ZIP4 was functionally involved in the cellular response to CDDP. Similarly, PEA2 cells were more resistant to CDDP than PEA1 cells (Figure 3E).

### 2.4. ZIP4 Represents a Highly Potent and Selective Marker for Tumor Formation In Vivo in HGSOC

**Figure 4:**
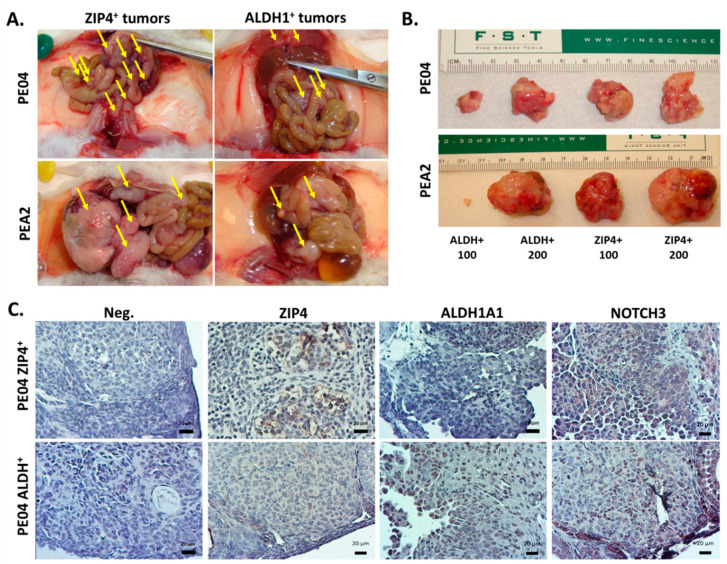
ZIP4^+^ cells exhibited higher tumor formation abilities compared to other groups of cells (**A**) Representative photographs of mice i.p. injected with PE04 ZIP4^+^, PE04 ALDH^+^, and PEA2 ZIP4^+^, cells with some tumor burdens are arrow-indicated. (**B**) Tumors generated from low numbers of ZIP4^+^ and ALDH^+^ cells (s.c. injected). (**C**) Representative photographs of IHC staining of ZIP4, ALDH1, and NOTCH3 in ZIP4^+^ and ALDH^+^ tumor sections. Scale Bar = 20 μm. The dark purple/brown or dark blue colored cells above the background are positive stained. Representative results are shown for more than three independent experiments.

Since the idea of CSC (or tumor initiating cells) is, in fact, more of an operational concept with high translational/clinical implications, we focused on testing the potential tumor forming capacity of ZIP4^+^ cells in vivo. ZIP4^+^ cells developed tumors and ascites in 47.3 ± 10.2 days (*N* = 7; Figure 4A and Table 1) when 10,000 PE04 ZIP4^+^ cells/mouse were i.p. injected; in contrast, injecting 10,000 ZIP4^−^ cells/mouse did not form tumors/ascites by day 267 ± 30 (*N* = 5) when the mice were sacrificed. The tumor nodules in ZIP4^+^ cells injected into mice were 249 ± 111 and the ascites volume/mouse averaged at 26.9 ± 14.5 mL (Table 1). Most (80%) tumor nodules were ≤1 mm^3^ with the remainder being 1–15 mm^3^. The common locations of these tumors were the diaphragm (D), fallopian tube (F), liver (L), mesentery (M), omentum (Om), ovary (Ov), pancreases (P), small intestine (SI), and peritoneal wall (PW; Table 1). These data indicate that ZIP4 is a potent and highly selective marker for tumor formation in HGSOC cells, since none of the ZIP4^−^ mice developed tumors/ascites over a long period of time (up to 267 ± 30 days).

Since ALDH activity is one of the most common and best-described CSC markers for EOC [3,6,7,40,41], we tested ALDH activity as a CSC marker in PE04 cells in comparison to ZIP4 under the same conditions. ALDH^+^ cell (10,000 cells/mouse) i.p. injected mice developed tumors and ascites in 91 ± 13 days (*N* = 5; Figure 4A and Table 1). The tumor nodules in ALDH^+^ cells injected mice were 95 ± 44 and the ascites volume/mouse averaged 14 ± 2.3 mL (Table 1). Most (80%) tumor nodules were ≤1 mm^3^ with the remainder being 1–15 mm^3^. The common locations of these tumors were the D, L, M, Ov, and PW (Table 1). In addition, while three of four ALDH^−^ cell injected mice did not develop any tumor/ascites by 220 days, one mouse developed tumors with 13 tumor nodules and 1.5 mL ascites. These results suggest that, as a single marker, ZIP4 is a more potent marker for in vivo tumor formation than ALDH activity in PE04 HGSOC cells.

We also tested ZIP4^+^ and ZIP4^−^ cells from PEA2 cells for their tumor formation capacity. As shown in Table 1, ZIP4^+^ cells (10,000 cell/mouse; i.p. injected) formed tumors and ascites in 102 ± 11 days (*N* = 5; with fewer but larger tumors than those from PE04 cells; Table 1). In contrast, the three ZIP4^−^ cells (10,000 cells/mouse) injected mice survived 238 days with only one of them developing tumors (Table 1).

Immunohistochemistry (IHC) staining showed that tumor sections from ZIP4^+^ cell-injected mice expressed high levels of ZIP4, ALDH1, and NOTCH3, and Ki67. On the contrary, ALDH^+^ tumors expressed ALDH1, but relatively low levels of ZIP4 and NOTCH3 (Figure 4B,C).

To test and validate ZIP4 as a CSC marker, we conducted limiting dilution experiments using ZIP4^+^ cells from both PE04 and PEA2 lines. FACS sorted ZIP4^+^ cells (100 or 200/s.c. site in mouse) were mixed with Matrigel and injected into mice subcutaneously. We also injected ALDH^+^ cells in the same manner for comparison. The data are summarized in Table 1 and shown in Figure 4B. PEA2 and PE04 ZIP4^+^ cell injected mice developed tumors, including those injected with as few as 100 ZIP4^+^ cells, at 81–110 days, respectively. In all cases, the tumor sizes derived from ZIP4^+^ cells were larger than those derived from the same numbers of ALDH^+^ cells from both PE04 and PEA2, supporting that ZIP4 was a more potent CSC marker in at least a subset of HGSOC cells.

Cell types and numbers injected, mouse number/group, survival days, ascites volumes, and tumor numbers and locations are summarized in the Table 1.

### 2.5. NOTCH3 Was an Important Down-Stream Mediator for ZIP4’s Activity

**Figure 5:**
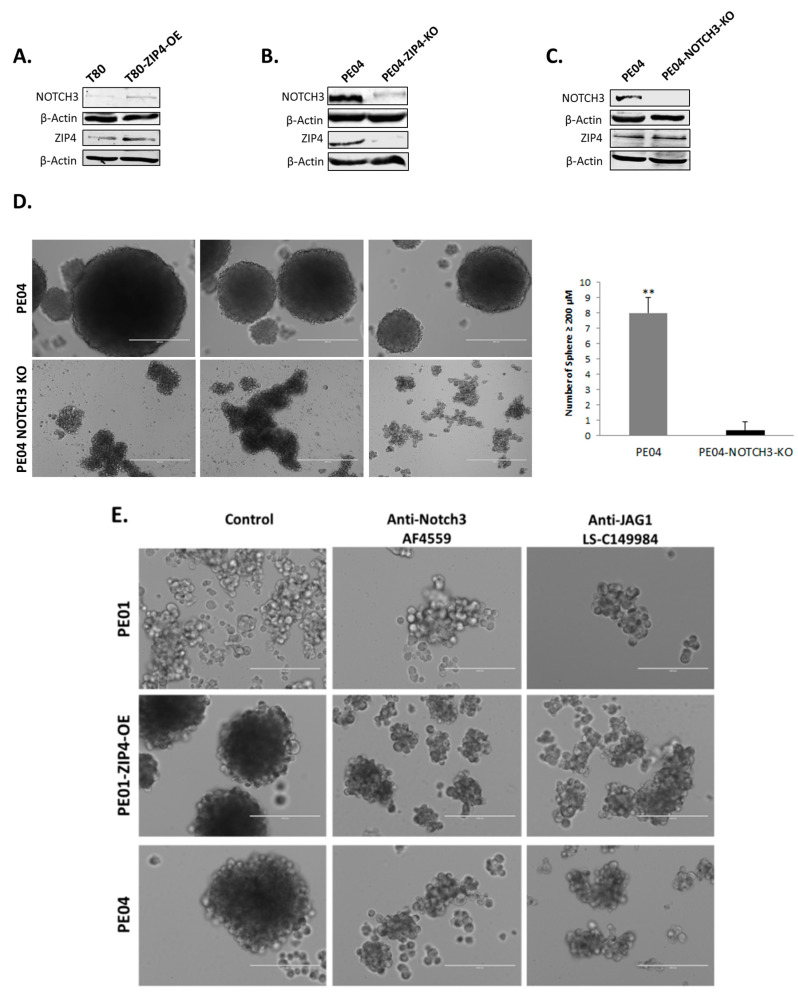

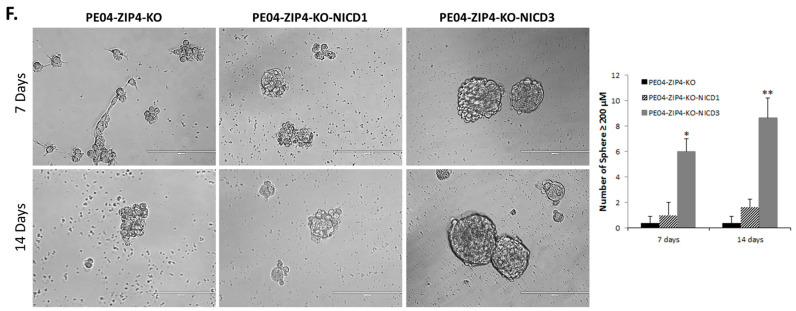
ZIP4 upregulated NOTCH3 expression and NOTCH3 was a functional downstream mediator of ZIP4. (**A**) Overexpression of ZIP4 increased NOTCH3 expression. (**B**) Knockout of ZIP4 reduced NOTCH3 expression. (**C**) Knockout of NOTCH3 in PE04 cells did not affect its ZIP4 expression. The uncropped Western blot images for (A) to (C) are shown in the Figure S2. (**D**) Knockout of NOTCH3 inhibited spheroid formation in PE04 cells. The bar graph depicts numbers of spheroid (≥200 μm) developed from PE04 and PE04-Notch3-KO from three independent studies. (**E**) Anti-NOTCH3 and anti-Jag1 neutralizing antibodies (0.25 UG/well) blocked the ability of spheroid formation in PE01-ZIP4-OE and PE04 cells, with 6 days of treatment. (**F**) NOTCH3 intracellular domain (NICD3), but not NICD1, reversed most spheroid-formation capacity in ZIP4-KO cells. The bar graph depicts numbers of spheroids (≥200 μm) formed from PE04-ZIP4-KO control, PE04-ZIP4-KO transfected with NICD1 or NICD3 in 7 days and 14 days, respectively (average ± SD). Representative results are shown for more than three independent experiments. Scale bar = 400 μm. * *p* < 0.05 and ** *p* < 0.01.

ZIP4, a zinc transporter, is not associated with enzymatic or transcription activity. How it regulates expression of other genes/proteins was the next question we considered. We postulated that at least part of its activities is mediated by interacting with other signaling module(s) with transcriptional activities. Since TCGA analyses of HGSOC tumors have revealed NOTCH activation/alteration as one of the four critical pathways altered in HGSOC [5], and it co-upregulated with ZIP4 in more aggressive vs. less aggressive mouse EOC cell pairs [15], we tested whether NOTCH1 and/or NOTCH3 could be the candidates. We found that ZIP4-OE in HOSE T80 cells induced NOTCH3, but not NOTCH 1 (Figure 5A and not shown). Conversely, ZIP4-KO diminished NOTCH3 expression in PE04 cells (Figure 5B).

To test whether NOTCH3 was functionally involved in the CSC-like activities in HGSOC cells, we generated NOTCH3-KO clones in PE04 cells using the CRISPR-Cas9 system and found that ZIP4 expression levels were not significantly changed in NOTCH3-KO cells (Figure 5C), suggesting that NOTCH3 was downstream of ZIP4. NOTCH3-KO completely blocked spheroid formation in PE04 cells (Figure 5D). Although irregular cell aggregations were observed in NOTCH3-KO cells, they never formed round spheroids with clear edges (Figure 5D). In addition, the neutralizing antibodies against either NOTCH3 or the NOTCH ligand Jagged-1 (Jag1) completely blocked spheroid formation in PE01-ZIP4-OE and PE04 cells (Figure 5E).

To further determine whether NOTCH3 acted as a functional important downstream mediator of ZIP4, we conducted complementary experiments by transfecting the active Notch intracellular domains of NOTCH1 and NOTCH3 (NICD1 and NICD3) into ZIP4-KO cells (Figure 5F). We found that NICD3, but not NICD1, reversed lost spheroid-formation capacity in ZIP4-KO cells, suggesting that NOTCH3 is a critical downstream mediator of ZIP4’s activity.

### 2.6. ZIP4 Physically Interacted with NOTCH3

**Figure 6:**
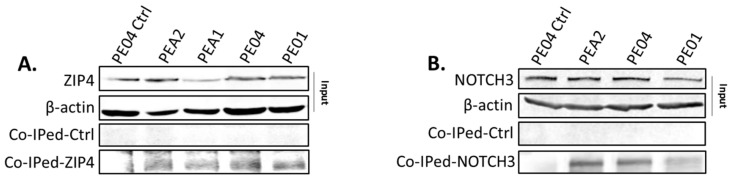
Physical interaction between ZIP4 and NOTCH3 (**A**) coimmunoprecipitations (IPs). The top rows: input before co-IP with β-actin as a loading control in the second row. The third and the fourth rows: Western blot to detect ZIP4. The third row shows results from the negative controls: first lane: no antibody bound to the bead and PE04 cell lysate was used; second to the fifth lanes: IgG (anti-human IgG antibody (ab109489, Abcam, Cambridge, MA, USA) was bound to beads and lysates from different cell lines were used. The fourth row shows the ZIP4 co-IPed by the NOTCH3 antibody from different cell lysates. (**B**) Reverse co-IP was performed by a ZIP4 antibody (20625-I-AP), followed by Western blot analysis to detect NOTCH3. The top two rows show the input with β-actin as a loading control in the second row. The third and the fourth rows: Western blot to detect NOTCH3. The third row shows the results from negative controls: first lane: no antibody bound to the bead and PE04 cell lysate was used; second to the fourth lanes: IgG was bound to breads, and lysates from different cell lines were used. The fourth row shows the NOTCH3 co-IPed by the ZIP4 antibody from different cell lysates. Pierce Cross-link IP Kit was used. Representative results are shown for more than three independent experiments. The uncropped Western blot images for (A) and (B) are shown in the Figure S2.

To test whether there was an interaction between ZIP4 and NOTCH3, a Pierce Cross-link IP Kit (Cat.Log# 26147) was used to conduct coimmunoprecipitation (co-IP) in PE01 and PE04 [34]. Using a NOTCH3 antibody (sc-5593, recognizing both the full length NOTCH3 and NOTCH3 NICD), ZIP4 was coimmunoprecipitated (Co-IPed) and detected by immunoblotting (Figure 6A). Reversed co-IP using a ZIP4 antibody (20625-1-AP) showed that NOTCH3 was co-IPed with ZIP4 (Figure 6B). On the other hand, two other related signaling molecules, NOTCH1 and β-catenin, did not interact with ZIP4 (not shown), providing evidence that the ZIP4-NOTCH3 interaction was specific.

### 2.7. NOTCH3-KO Significantly Reduced Tumorigenesis In Vivo

**Figure 7:**
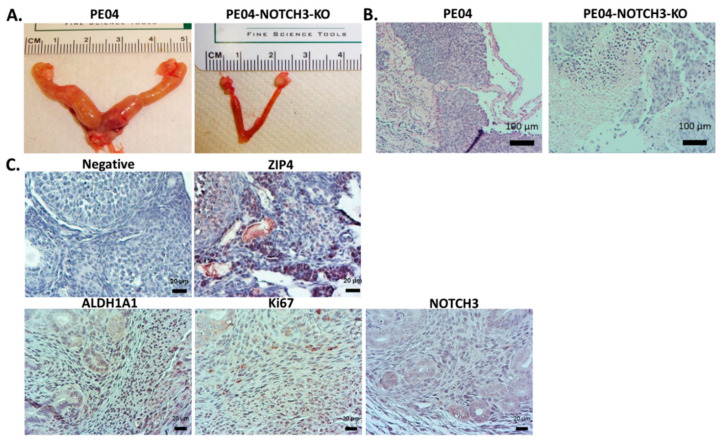
NOTCH3 knockout reduced tumorigenesis in NSG mice. (**A**) Representative tumors formed in PE04 vs. PE04-NOTCH3-KO injected mice. (**B**) Representative H&E staining of tumors formed in PE04 and PE04-NOTCH3-KO injected NSG mice. (**C**) Representative IHC staining for ZIP4, ALDH1A1, Ki67, and NOTCH3 in NOTCH3-KO tumor section. Samples without the first antibody were used as negative staining controls. The dark purple/brown or dark blue colored cells above the background are positive stained.

PE-04 cells (5 × 10^6^ cell/mouse; i.p. injected) formed tumors/ascites in 42 days (Table 1). PE04-ZIP4-KO significantly delayed tumor development as we reported previously [14]. We tested the effect of NOTCH3-KO on tumorigenesis. NOTCH3-KO (PE04-NOTCH3-KO cells 5 × 10^6^ cell/mouse; i.p. injected) greatly reduced tumorigenesis by extending mouse survival to 161 ± 21 days, with tumor numbers and ascites volumes significantly lower than the control mice (Figure 7A and Table 1). These data indicate the importance of NOTCH3 in tumorigenesis. As shown in Figure 7B, NOTCH3-KO tumors expressed ALDH1 and Ki67, but not NOTCH3.

## 3. Discussion

We identified ZIP4 as a novel CSC marker in HGSOC using PE04 and PEA2 and PDX cells. In vitro, ZIP4 is expressed at high levels in spheroids, functionally involved in spheroid formation, and converts resistance to CDDP. ZIP4^+^ cells have self-renewal and differentiation abilities. In vivo, injection of as few as 100 ZIP4^+^ cells from PE04 and PEA2 HGSOC lines formed tumors in all mice injected. In particular, when compared to the extensively characterized EOC CSC marker ALDH, ZIP4 shows higher potency in tumor formation, as measured by tumor/ascites formation time, tumor size, and/or extent, and uptake rate. We noticed that the HGSOC cell lines/PDX tested in this work contained higher percentages of ZIP4^+^ than ALDH^+^ cells (2.8–10.0% vs. 1–6% for ZIP4 and ALDH, respectively). These data support our finding that ZIP4 is a new potent CSC marker in at least a subset of HGSOC. While ZIP4’s tumor promoting roles have been reported in several cancers [9,10,11,12,13], its CSC marker value, reported for the first time here, in additional cancers is an interesting area to be studied in the future.

Our collective in vitro and in vivo data showed that ZIP4 is not only a marker, but also a therapeutic target for HGSOC. Although many CSC markers have been previously identified, the functional roles of several of them are not well characterized. In addition, whether these markers are drivers or passengers is largely unknown. In contrast, we provided evidence that ZIP4 is not only functionally involved in CSC activities as measured by drug-resistance, spheroid and colony formation in vitro, and tumor formation in vivo, but also an upstream regulator of several known CSC markers, including ALDH1, OCT4 [14], SOX9 [14,15,16], and NOTCH3, making ZIP4 a more logical and potentially more effective target for HGSOC.

Among the zinc transporters (14 ZIP and 10 ZnT genes/proteins) studied in cancers, many of them had decreased expression in tumors vs. normal tissues or have increased expression in only certain subtypes of cancer or cell lines, with only one to two reports for that specific Zip or ZnT [42]. Thus, ZIP4 stands out as an important oncogene and target. Currently, a selective inhibitor for ZIP4 has yet to be developed. However, this task is conceptually possible, since a specific inhibitor against another ZIP family member, ZIP7, already has been developed [43]. Moreover, development of a more effective delivery of siRNAs is a very active field in ovarian cancer research [44], with high promise for genetically targeting ZIP4 in the future. Manipulating extracellular zinc may be an alternative approach. Extracellular zinc-dependent ZIP4 actions have been extensively studied in physiology and cancers [45,46,47]. However, we have shown that ZIP4 may also have extracellular zinc-independent actions [14]. In addition, zinc is an essential element, playing critical roles in both physiology and pathology [42]. Zinc not only constitutes a structural element for more than 3000 proteins but also plays important regulatory functions in cellular signal transduction [42]. Hence, it is not surprising that multiple markers/pathways can be regulated by ZIP4. Hence, it is anticipated that inhibiting zinc using zinc chelators in vivo is likely to generate complex reactions and toxicity. At any rate, our current work was a significant step forward to our understanding of these complex regulations.

The expression of NOTCH and/or its ligands is regulated by several known factors, including, but not limited to, the F-box with 7 tandem WD40 (FBXW7) protein and transcription factors binds to antioxidant response elements (AREs), and epigenetic regulating factors and microRNAs [48,49,50,51,52,53]. We show, for the first time, that NOTCH3 was regulated by ZIP4 in PE04 HGSOC cells. ZIP4 was not a transcription factor. Our co-IP data suggest that the direct interaction between ZIP4 and NOTCH3 may provide a mechanism by which ZIP4 stabilizes and/or activates NOTCH3, which remains to be further investigated.

Importantly, we found that NOTCH3 is a critical down-stream functional mediator of ZIP4’s spheroid-formation activity in vitro. Spheroid-formation has now been widely accepted as a standard assay for CSC activity in vitro [37,38,54,55]. Our data suggested that the ZIP4-NOTCH3 axis played an important role in CSC in HGSOC cells. We observed that NOTCH3-KO cells never formed round spheroids with clear edges, although they did form some cell aggregates (Figure 5D). These aggregates are loosely packed and lack not only true spherical geometry, but possibly also cell–cell and cell–matrix interactions, impacting biological properties as indicated [56,57]. However, their differences at the molecular level are still poorly understood and remain to be further elucidated. The role NOTCH3 plays in the transition from aggregates to spheroids may lead to interesting findings in this area.

The importance of NOTCH signaling in cancer, and in CSC in particular, has been well-recognized [27,52,58,59,60,61,62]. Overexpression, gene amplification, and abnormal activation of NOTCH3 are associated with different cancers including ovarian cancer [5,52]. NOTCH3 overexpression is related to the recurrence of ovarian cancer and confers resistance to carboplatin [24]. While the majority of in vivo NOTCH3 studies in ovarian cancer testing the NOTCH3 effects have used activated NOTCH3 or its regulators, and γ-secretase inhibitors [52,63,64,65], Hu et al. have shown that silencing NOTCH3 with siRNA in a combination with paclitaxel reduced tumor proliferation and angiogenesis in SKOV3, A2780, and OVCAR5 cells [60]. Among these cell lines, only OVCAR5 may represent HGSOC [28,66,67] and siRNA-mediated silencing is incomplete. We generated a CRISPR-mediated and more complete NOTCH3 knockout in the HGSOC PE04 cell line and shown here that NOTCH3-KO has dramatically delayed tumor development in vivo, supporting the importance of NOTCH3 in tumorigenesis in ovarian cancer. In addition, targeting NOTCH3, but not NOTCH1/2 may represent a critical advantage. Among the four NOTCH receptors (NOTCH1-4), NOTCH1/2 have been considered to be the major NOTCH receptors involved in normal physiology [68,69]. In addition, both NOTCH1/2 knockout cause embryonically lethality in mice, while NOTCH3 knockout only generates minor postnatal defects [70,71,72]. These data suggest that targeting NOTCH3, rather than NOTCH1/2, may generate less toxicity. Inhibition of NOTCH signaling in combination with paclitaxel reduces platinum-resistant ovarian tumor growth [48,59,60,61]. However, while γ-secretase-based preclinical and/or clinical trials are promising, there are still considerable issues with efficacy and/or off-target toxicity [73,74,75,76,77]. The new ZIP4-NOTCH3 axis provides a potential new way to block NOTCH signaling.

ZIP4 has been shown to have tumor promoting activities in several cancers [9,10,11,12,13], with a broad range of activities, including promoting resistance to apoptosis, cell proliferation, invasion, epithelial-to-mesenchymal transition, cytokine secretions, upreregulation of oncogenes, and tumor progression [9,10,12,13,47,78]. It is very possible that not all the functions of ZIP4 are mediated by NOTCH3, which needs to be further investigated. Similarly, NOTCH3 is regulated by many different factors [48,49,50,51,52,53] and it interacts with many other oncogenes [27,58,59,61,62]. It is almost certain that NOTCH3 has ZIP4-independent activities. In fact, it is very unlikely that any one oncogene is regulated by a single pathway or its role is mediated by a single mediator. These concepts are clearly supported by the large body of bioinformatics studies in recent decades. However, none of these studies diminishes the high significance of experimental studies revealing new links and/or nodes in oncogenic studies.

## 4. Materials and Methods

### 4.1. Reagents, Cell Lines, and Culture

For Western blot and immunohistochemistry (IHC) staining, an antihuman ZIP4 antibody (20625-1-AP; Proteintech, Rosemont, IL, USA) was used. For FACS assay and sorting, an antimouse ZIP4 antibody (AF7315; R&D Systems, Minneapolis, MN, USA) was used. Anti-NOTCH1 (SC-23304, Santa Cruz Biotechnology, Dallas, TX, USA), anti-NOTCH3 (sc-5593; Santa Cruz Biotechnology, Dallas, TX, USA), anti-NOTCH3 (ab23426; Abcam, Cambridge, MA, USA), and anti-ALDH1A1 (SAB1403542, Sigma-Aldrich, St. Louis, MO, USA) were from the companies as indicated. The pair of PE01/PE04 cell lines was from Dr. Daniela Matei (Northwestern University); the pairs of PEA1/PEA2 cells were from Sigma (St Louis, MO, USA); and the OVCAR3 cells were obtained from ATCC (Manassas, VA, USA). The Kuramochi cells were from Dr. Anirban Mitra (Indiana University). The T29 and T80 human ovarian surface epithelial cell lines were from Dr. Jinsong Liu (M.D. Anderson). The human fallopian tube FT33-R24C and FT194 cell lines were from Dr. Ronny Drapkin (UPenn). PE04-ZIP4-KO cell lines were generated using CRISPR as we described previously [14]. PE04-NOTCH3-KO cell line was generated by infecting CRISPR lentivirus vectors (HCP211876-LvSG03-3-B, GeneCopoeia, Rockville, MD, USA) three times and stable clones were selected by puromycin (0.5 μg/mL). All cell lines were maintained in a humidified atmosphere at 37 °C with 5% CO_2_. OVCAR3 cells were maintained in RPMI-1640 supplemented with 20% FBS (ATCC, Manassas, VA, USA), 0.01 mg/mL insulin, 50 U/mL penicillin, and 50 μg/mL streptomycin. PE01/PE04 cells were cultured in RPMI 1640 with glutamine, 10% fetal bovine serum (FBS), and 100 μg/mL penicillin-streptomycin-amphotericin B. PEA1 and PEA2 were cultured in RPMI 1640 with 2 mM glutamine, 2 mM sodium pyruvate, 10% FBS, and 100 μg/mL penicillin-streptomycin-amphotericin. Basement membrane matrix (Matrigel) was from BD Biosciences (Bedford, MA, USA).

### 4.2. The Patient-Derived Xenograft (PDX) Model and Human HGSOC Tissues

The PDX line was from the ascites of a patient with a stage III serous adenocarcinoma collected at the time of surgery at Indiana University Hospital, Indianapolis, USA, under IRB approval. Patient-derived xenograft (PDX) models were generated from tumor cells isolated from the ascites: 50 mL fresh ascites from the patient were centrifuged at 3000 rpm for 10 min. The pellet was mixed with 10 mL 1X RBC lysis buffer for 10 min. at room temperature, filtered (40-μm cell strainer) and washed twice with PBS. RBCs were removed by Histopaque-1077 (MilliporeSigma, St. Louis, MO, USA). The resulting single tumor cells were placed under stem cell conditions by resuspension in serum-free DMEM/F12 supplemented with 5 μg/mL insulin (MilliporeSigma, St. Louis, MO, USA), 20 ng/mL human recombinant epidermal growth factor (EGF; Thermo Fisher Scientific corporation, Waltham, MA, USA),10 ng/mL basic fibroblast growth factor (bFGF; Thermo Fisher Scientific corporation), and 0.4% bovine serum albumin (BSA; Sigma), followed by culturing in ultra low attachment plates (Corning, Corning, NY, USA) and subsequent organization into spheroids. The PDX line was propagated in NSG mice 3 times by i.p. injection, including once with fresh tumor cells and twice with spheroid cells. Tumors formed 90 days after injection and we collected ascites and tumor tissues from two NSG mice. Ovarian carcinoma tissues were minced, suspended in DMEM/F12 medium (Invitrogen, Carlsbad, CA, USA), mixed with 300 units/mL of both collagenase (Invitrogen) and hyaluronidase (Calbiochem, San Diego, CA, USA), and followed by overnight incubation (37 °C, 5% CO_2_). After enzymatic disaggregation, cells were harvested for experiments. Human HGSOC tissues were obtained from CHTN as we described in our previous studies through Dr. Xu’s IRB [79].

### 4.3. Western Blot Analysis

Western blot analyses were conducted using standard procedures and proteins were detected using primary antibodies and fluorescent secondary antibodies (IRDye 800CW-conjugated or IRDye 680-conjugated antispecies IgG, Li-Cor Biosciences, Lincoln, NE, USA) as we described previously [2].

### 4.4. mRNA Expression Analyses Using Quantitative-PCR (Q-PCR)

The same numbers of cells (5000/well into 6-well) were initially seeded for 3D (Costar^®^ 6-well Ultra-Low Attachment Plates) and 2D cultures. We collected cells after 3 and 14 days for the 2D and 3D cultures, respectively. Q-PCR analyses were conducted to examine ZIP4 mRNA levels in HGSOC cell lines when cultured as spheroids vs. in 2D dishes. The mRNA levels of GAPDH were used as internal controls of each sample. ZIP4 expression levels as ratios to those of GAPDH in each cell line were calculated and the ratio in the COVAR3 was arbitrarily chosen as 1.0. Primer pairs used in this study were: GAPDH: F, 5′-CACCATTGGCAATGAGCGGTTC-3′/R, 5′-AGGTCTTTGCGGATGTCCACGT-3′ and ZIP4: F, 5′-ATGTCAGGAGCGGGTCTTGC-3′/R, 5′-GCTGCTGTGCTGCTGGAAC-3′.

### 4.5. Assessments of Spheroid Renewal and Differentiation

A similar method as described by Zhang et al. [38] was employed to examine tumor-like epithelial differentiation of anchorage-independent cells. A portion of spheroids was dissociated by trypsin and single cells were plated in 2D dishes under standard differentiating conditions (DMEM/F12 supplemented with 10% fetal bovine serum (FBS) without growth factors). The rest of the spheroids were passaged four times. The expression and ZIP4 and ALDH1A were examined in both the 4th passaged spheroids and in 2D-cultured cells.

### 4.6. Coimmunoprecipitation (Co-IP)

Coimmunoprecipitations (IP) were performed according to the instructions in the Pierce Cross-link IP Kit (Prod# 26147) using control IgG (Anti-Human IgG antibody (ab109489, Abcam, Cambridge, MA, USA), ZIP4 antibody (20625-1-AP, Proteintech Group, Rosemont, IL, USA), or NOTCH3 antibody (ab23426, Abcam, Cambridge, MA, USA) as the bait.

### 4.7. Spheroids and Colony Formation Assay

Cancer cells were trypsinized using 0.25% trypsin and washed twice in PBS in order to prepare single-cell suspensions. Spheroids were photographed after seven days in culture. Two thousand cancer cells per well were seeded into ultra-low-attachment 96-well plates containing 200 μL of the DMEM/F12 supplemented with 5 μg/mL insulin (MilliporeSigma, St. Louis, MO, USA), 20 ng/mL human recombinant epidermal growth factor (EGF; Thermo Fisher Scientific corporation, 10 ng/mL basic fibroblast growth factor (bFGF; Thermo Fisher Scientific corporation, Waltham, MA, USA), and 0.4% bovine serum albumin (BSA; Sigma), followed by culturing in 24- or 96-well ultra low attachment plates (Corning, Corning, NY, USA). Spheroids were photographed after 14 days in culture. For colony formation assays, cells were seeded into 6-cm^2^ plates at a cell density of 1000 cells per plate. The colonies were counted after staining with crystal violet dye (Sigma) at day 10.

### 4.8. Cytotoxicity Assay

Cell viability was determined by measuring cell metabolic activity using the MTT assay. Cells were seeded at 2000/well in 96-well plates and then exposed to varying concentrations of cisplatin (CDDP; BD Biosciences, San Jose, CA, USA) 24 h post-seeding for 48 and 72 h. Each group was replicated in three separate wells. Cell survival was measured using MTT as described previously [14].

### 4.9. Fluorescence-Activated Cell Sorting (FACS) Analysis and the ZIP4^+^ Cell Self-Renewal Assay

Human PE01, PE04, PEA1, PEA2, and Kuramochi cells were detached and labeled by the ZIP4 antibody (AF7315; R&D Systems, 1:50) at room temperature for 2 h, followed by incubation with a APC-labeled donkey anti-goat IgG secondary antibody (F0108; R&D Systems, 1:500). ALDH activity was measured using the ALDEFLOUR Kit (STEMCELL Technologies, Vancouver, Canada) as we described previously [15] and detected using the green fluorescence channel (520–540 nm). FACS-based sorting and analysis of markers ZIP4 and ALDH were conducted using the BD FACSAria cell sorter system (Becton-Dickinson, Franklin Lakes, NJ, USA) and BD LSR Fortessa Analyzer (BD Biosciences, San Jose, CA, USA), and data analyzed by FlowJo V10 (BD Biosciences, San Jose, CA, USA). For the self-renewal assay, FACS-sorted ZIP4^+^ cells (100% pure) were collected and seeded into triple wells (1 × 10^4^ cells per well), cultured under differentiation conditions (DMEM medium with 10% FBS) for 48 h, trypsinized using 0.25% trypsin and washed twice in PBS, then cells were relabeled with ZIP4/APC antibodies before the renew/differentiation assays.

### 4.10. Xenograft Mouse Model

All animal experiments were done under the protocols approved by Indiana University Animal Care and Use Committee. The protocol # was 11345, which complies with the ARRIVE guidelines, with details of the study design, experimental, monitoring, and outcome measurement procedures, and justified the sample size (*N* = 4–7 for our different mouse groups in this study). This information and data were also provided in this manuscript.

ZIP4^+^ and ZIP4^−^ groups served as controls to each other and quantitative and repetitive results are shown in the manuscript. In addition, the mice in different groups served as controls to each other. For example, the ZIP4^+^ group was the control for both ZIP4^−^ and ALDH^+^ groups.

Female NSG mice were obtained from the In Vivo Therapeutics Core, Indiana University School of Medicine (Indianapolis, IN). At 6–8 weeks of age, different cell populations from PE04 (ZIP4^+^, ZIP4^−^, ALDH^+^, and ALDH^−^, 10,000 cells in 200 μL of DMEM/mouse) were i.p. injected into mice. In addition, dissociated PE04 spheroid cells (1000–2000 cells/mouse) were counted, resuspended in 200 μL DMEM containing 10% FBS, and injected i.p. into NSG mice.

Engrafted mice were inspected daily for tumor/ascites appearance by visual observation, palpation, and tumor latencies. Mice were sacrificed by cervical dislocation at a tumor diameter of 1 cm or at 270 days post-transplantation. Tumors were counted at each metastatic location, with tumor diameters were measured. Animal protocols were approved by the Indiana University School of Medicine Animal Care and Use Committee (#11345). Xenograft tumors were resected, fixed in 10% neutral, buffered formalin, and embedded in paraffin for sectioning (5 μm) on a rotary microtome, followed by slide mounting; H&E staining and histologic assessment was conducted by Dr. Robert Emerson, a pathologist at the Indiana University School of Medicine.

For limiting dilution experiments, subcutaneous (s.c) injections were used. A total of 100/200 ZIP4^+^ or ALDH1^+^ FACS sorted cells in DMEM with 10% FBS were mixed with the Matrigel Basement Membrane Matrix (BD Biosciences, Bedford, MA, USA; ratio, 1:1). The mixture (0.1 mL) was injected subcutaneously into 6–8 week old NSG female mice to the front, left, rear left, front right, or rear right flank. Engrafted mice were monitored biweekly for tumor development by visual observation and palpation. Mice were sacrificed by cervical dislocation at a tumor diameter of 1 cm. The tumor sizes were measured with digital calipers and calculated as follows: tumor size (cm^3^) = [width (cm)]2 × [length (cm)] × π/6.

### 4.11. Statistical Analyses

The Student’s *t*-test was utilized to assess the statistical significance of the difference between two treatments. The asterisk rating system as well as quoting the *p* value in this study was * *p* < 0.05; ** *p* < 0.01; and *** *p* < 0.001. A *p* value of less than 0.05 was considered significant.

## 5. Conclusions

We presented data to show that ZIP4 is a novel CSC marker and target in HGSOC. In addition, we revealed a novel ZIP4-NOTCH3 axis involved in CSC-like activities, including forming spheroid and colonies in vitro and tumor/ascites in vivo. The major highlights of the current work include that (1) ZIP4 is a novel CSC marker in HGSOC; (2) ZIP4 is a more potent CSC marker than the most extensively characterized ovarian cancer stem cell marker, ALDH, in at least a subset HGSOC; (3) ZIP4 is an upstream regulator of another CSC-marker, NOTCH3, and it physically binds to NOTCH3; (4) NOTCH3 is functionally involved in spheroid formation in vitro and tumorigenesis in vivo in HGSOC; and (5) our data suggest that ZIP4 and the novel ZIP4-NOTCH3 axis represent important therapeutic targets in HGSOC.

Taken together, our data presented here likely make a significant impact on developing new scientific concepts for ZIP4-related cell signaling and functional involvement in HGSOC, which may be expanded beyond EOC. In addition, the data provided bases to develop mechanism-based targeting strategies in EOC.

## Figures and Tables

**Table 1:** Summary of tumor formation abilities of different groups of cells in vivo.

**Cell Type**	**Injection Site**	**Cell No.**	**Mice No.**	**Survival Days**	**Ascites Vol. (mL)**	**Tumor No.**	**Tumor Sites**
PE04 ZIP4^+^	I.P.	10K	7/7	47 ± 10	26.90 ± 14.55	249 ± 111	D, F, L, M, Om, Ov, P, SI, PW
PE04 ZIP4^−^	I.P.	10K	0/5	267 ± 30	0.00	0	NONE
PE04 ALDH^+^	I.P.	10K	5/5	91 ± 13	14.00 ± 2.35	95 ± 44	D, F, L, M, Ov, PW
PE04 ALDH^-^	I.P.	10K	1/4	217	1.50	13	D, W
PEA2 ZIP4^+^	I.P.	10K	5/5	102 ± 11	1.00 ± 0.60	17 ± 4	Ov, M, P, SI, PW, L
PEA2 ZIP4^−^	I.P.	10K	1/3	238	1.00	12	PW, M
**Cell Type**	**Injection Site**	**Cell No.**	**Mice No.**	**Survival Days**	**Tumor Size (cm^3^)**	**Tumor No.**	**Tumor Sites**
PE04 ZIP4^+^	S.C.	100	3/4	110 ± 5	0.17 ± 0.16	1	Front Left
PE04 ZIP4^+^	S.C.	200	4/4	110 ± 5	2.24 ± 1.13	1	Rear Left
PE04 ALDH^+^	S.C.	100	2/3	110 ± 5	0.04 ± 0.06	1	Front Right
PE04 ALDH^+^	S.C.	200	3/3	110 ± 5	1.41 ± 0.76	1	Rear Right
PEA2 ZIP4^+^	S.C.	100	3/3	81 ± 21	2.30 ± 3.75	1	Front Left
PEA2 ZIP4^+^	S.C.	200	3/3	81 ± 21	5.85 ± 5.20	1	Rear Left
PEA2 ALDH^+^	S.C.	100	3/3	81 ± 21	0.02 ± 0.02	1	Front Right
PEA2 ALDH^+^	S.C.	200	3/3	81 ± 21	3.14 ± 5.12	1	Rear Right
**Cell Type**	**Injection Site**	**Cell No.**	**Mice No.**	**Survival Days**	**Ascites Vol. (mL)**	**Tumor No.**	**Tumor Sites**
PE04	I.P.	5 × 10^6^	5/5	42 ± 4	12.10 ± 3.25	137 ± 27	D, M, Om, Ov, P, SI, PW
PE04-NOTCH3-KO	I.P.	5 × 10^6^	6/6	161 ± 21	4.72 ± 1.53	36 ± 18	D, M, Ov, PW

D, diaphragm; F, Fallopian tube; L, liver; M, mesentery; Om, omentum; Ov, Ovary; P, pancreases; SI, small intestine; PW, peritoneal wall.

## Supplementary Materials

The following are available online at https://www.mdpi.com/2072-6694/12/12/3692/s1, Figure S1: Representative photographs of tumors formed in Patient-Derived Xenograft model. Figure S2. Uncropped western blots.

## References

[1] Rojas V., Hirshfield K.M., Ganesan S., Rodriguez-R L. (2016). Molecular characterization of epithelial ovarian cancer: Implications for diagnosis and treatment. Int. J. Mol. Sci..

[2] Klemba A., Purzycka-Olewiecka J.K., Wcisło G., Czarnecka A.M., Lewicki S., Lesyng B., Szczylik C., Kieda C. (2018). Surface markers of cancer stem-like cells of ovarian cancer and their clinical relevance. Contemp. Oncol..

[3] Nwani N.G., Condello S., Wang Y., Swetzig W.M., Barber E., Hurley T., Matei D. (2019). A novel ALDH1A1 inhibitor targets cells with stem cell characteristics in ovarian cancer. Cancers.

[4] Reya T., Morrison S.J., Clarke M.F., Weissman I.L. (2001). Stem cells, cancer, and cancer stem cells. Nature.

[5] The Cancer Genome Atlas Research Network (2011). Integrated genomic analyses of ovarian carcinoma. Nature.

[6] Keyvani V., Farshchian M., Esmaeili S.-A., Hadi Y., Moghbeli M., Nezhad S.-R.K., Abbaszadegan M.R. (2019). Ovarian cancer stem cells and targeted therapy. J. Ovarian Res..

[7] Al-Alem L.F., Pandya U.M., Baker A.T., Bellio C., Zarrella B.D., Clark J., DiGloria C.M., Rueda B.R. (2019). Ovarian cancer stem cells: What progress have we made?. Int. J. Biochem. Cell Biol..

[8] Kambe T., Hashimoto A., Fujimoto S. (2014). Current understanding of ZIP and ZnT zinc transporters in human health and diseases. Cell Mol. Life Sci..

[9] Donahue T., Hines O.J. (2010). The ZIP4 pathway in pancreatic cancer. Cancer Biol. Ther..

[10] Li M., Zhang Y., Liu Z., Bharadwaj U., Wang H., Wang X., Zhang S., Liuzzi J.P., Shou-Mei Chang S.-M. (2007). Aberrant expression of zinc transporter ZIP4 (SLC39A4) significantly contributes to human pancreatic cancer pathogenesis and progression. Proc. Natl. Acad. Sci. USA.

[11] Lin Y., Chen Y., Wang Y., Yang J., Zhu V.F., Liu Y., Cui X., Chen L., Yan W., Jiang T. (2013). ZIP4 is a novel molecular marker for glioma. Neuro. Oncol..

[12] Xu X., Guo H.-J., Xie H.-J., Li J., Zhuang R.-Z., Ling Q., Zhou L., Wei X.-Y., Liu Z.-K., Ding S.-M. (2014). ZIP4, a novel determinant of tumor invasion in hepatocellular carcinoma, contributes to tumor recurrence after liver transplantation. Int. J. Biol. Sci..

[13] Zeng Q., Liu Y.-M., Liu J., Han J., Guo J.-X., Lu S., Huang X.-M., Yi P., Lang J.-Y. (2019). Inhibition of ZIP4 reverses epithelial-to-mesenchymal transition and enhances the radiosensitivity in human nasopharyngeal carcinoma cells. Cell Death Dis..

[14] Fan Q., Cai Q., Li P., Wang W., Wang J., Gerry E., Wang T.-L., Shih I.-M., Nephew K.P., Xu Y. (2017). The novel ZIP4 regulation and its role in ovarian cancer. Oncotarget.

[15] Cai Q., Fan Q., Buechlein A., Miller D., Nephew K.P., Liu S., Wan J., Xu Y. (2018). Changes in mRNA/protein expression and signaling pathways in in vivo passaged mouse ovarian cancer cells. PLoS ONE.

[16] Fan Q., Cai Q., Xu Y. (2017). LPA regulates SOX9 in ovarian cancer cells. J. Obstetrics Gynecolog..

[17] Wu D.M., Liu T., Deng S.-H., Han R., Xu Y. (2017). SLC39A4 expression is associated with enhanced cell migration, cisplatin resistance, and poor survival in non-small cell lung cancer. Sci. Rep..

[18] Braune E.B., Lendahl U., Notch L. (2016). A goldilocks signaling pathway in disease and cancer therapy. Discov. Med..

[19] Ivan C., Hu W., Bottsford-Miller J., Zand B., Dalton J.J., Liu T., Huang J., Nick A.M., Lopez-Berestein G., Coleman R.L. (2013). Epigenetic analysis of the Notch superfamily in high-grade serous ovarian cancer. Gynecol. Oncol..

[20] Lai I.C., Shih P.-H., Yao C.-J., Yeh C.-T., Wang-Peng J., Lui T.-N., Chuang S.-E., Hu T.-S., Lai T.-Y., Lai G.-M. (2015). Elimination of cancer stem-like cells and potentiation of temozolomide sensitivity by Honokiol in glioblastoma multiforme cells. PLoS ONE.

[21] Park J.T., Li M., Nakayama K., Mao T.-L., Davidson B., Zhang Z., Kurman R.J., Eberhart C.G., Shih I.-M., Wang T.-L. (2006). Notch3 gene amplification in ovarian cancer. Cancer Res..

[22] Choi J.H., Joon T., Park J.T., Davidson B., Morin P.J., Shih I.-M., Wang T.-L. (2008). Jagged-1 and Notch3 juxtacrine loop regulates ovarian tumor growth and adhesion. Cancer Res..

[23] Chen X., Stoeck A., Lee S.J., Shih I.-M., Wang M.M., Wang T.-L. (2010). Jagged1 expression regulated by Notch3 and Wnt/beta-catenin signaling pathways in ovarian cancer. Oncotarget.

[24] Park J.T., Chen X., Tropè C.G., Davidson B., Shih I.-M., Wanget T.-L. (2010). Notch3 overexpression is related to the recurrence of ovarian cancer and confers resistance to carboplatin. Am. J. Pathol..

[25] Jung S.G., Kwon Y.D., Song J.A., Back M.J., Lee S.Y., Lee C., Hwang Y.Y., An H.J. (2010). Prognostic significance of Notch 3 gene expression in ovarian serous carcinoma. Cancer Sci..

[26] Rahman M.T., Nakayama K., Rahman M., Katagiri H., Katagiri A., Ishibashi T., Ishikawa M., Iida K., Nakayama S., Otsuki Y. (2012). Notch3 overexpression as potential therapeutic target in advanced stage chemoresistant ovarian cancer. Am. J. Clin. Pathol..

[27] Ceccarelli S., Megiorni F., Bellavia D., Marchese C., Screpanti I., Checquolo S. (2019). Notch3 targeting: A novel weapon against ovarian cancer stem cells. Stem. Cells Int..

[28] Domcke S., Sinha R., Levine D.A., Sander C., Schultz N. (2013). Evaluating cell lines as tumour models by comparison of genomic profiles. Nat. Commun..

[29] Thu K.L., Papari-Zareei M., Stastny V., Song K., Peyton M., Martinez V.D., Zhang Y.-A., Castro I.B., Varella-Garcia M., Liang H. (2017). A comprehensively characterized cell line panel highly representative of clinical ovarian high-grade serous carcinomas. Oncotarget.

[30] Elias K.M., Emori M.M., Papp E., MacDuffie E., Konecny G.E., Velculescu V.E., Drapkin R. (2015). Beyond genomics: Critical evaluation of cell line utility for ovarian cancer research. Gynecol Oncol..

[31] Stronach E.A., Alfraidi A., Rama N., Datler C., Studd J.B., Agarwal E., Guney T.G., Charlie Gourley C., Hennessy B.T., Mills G.B. (2011). HDAC4-regulated STAT1 activation mediates platinum resistance in ovarian cancer. Cancer Res..

[32] Ricci F., Bizzaro F., Cesca M., Guffanti F., Ganzinelli M., Decio A., Ghilardi C., Perego P., Fruscio R., Buda A. (2014). Patient-derived ovarian tumor xenografts recapitulate human clinicopathology and genetic alterations. Cancer Res..

[33] Liu J.F., Palakurthi S., Zeng Q., Zhou S., Ivanova E., Huang W., Zervantonakis J.K., Selfors L.M., Shen Y., Pritchard C.C. (2017). Establishment of patient-derived tumor xenograft models of epithelial ovarian cancer for preclinical evaluation of novel therapeutics. Clin. Cancer Res..

[34] Lewis A.D., Hayes J.D., Wolf C.R. (1988). Glutathione and glutathione-dependent enzymes in ovarian adenocarcinoma cell lines derived from a patient before and after the onset of drug resistance: Intrinsic differences and cell cycle effects. Carcinogenesis.

[35] Sakai W., Swisher E.M., Jacquemont C., Chandramohan K.V., Couch F.J., Langdon S.P., Wurz K., Higgins J., Villegas E., Taniguchi T. (2009). Functional restoration of BRCA2 protein by secondary BRCA2 mutations in BRCA2-mutated ovarian carcinoma. Cancer Res..

[36] Muralikrishnan V., Hurley T.D., Nephew K.P. (2020). Targeting aldehyde dehydrogenases to eliminate cancer stem cells in gynecologic malignancies. Cancers.

[37] Wang Y., Zong X., Mitra S., Mitra A.K., Matei D., Nephew K.P. (2018). IL-6 mediates platinum-induced enrichment of ovarian cancer stem cells. JCI Insight.

[38] Zhang S., Balch C., Chan M.W., Lai H.-C., Matei D., Schilder J.M., Yan P.S., Huang T.H.-M., Nephewet K.P. (2008). Identification and characterization of ovarian cancer-initiating cells from primary human tumors. Cancer Res..

[39] Najafi M., Mortezaee K., Majidpoor J. (2019). Cancer stem cell (CSC) resistance drivers. Life Sci..

[40] Zhang T., Xu J., Deng S., Zhou F., Li J., Zhang L., Li L., Wang Q.-E., Fuhai Li F. (2018). Core signaling pathways in ovarian cancer stem cell revealed by integrative analysis of multi-marker genomics data. PLoS ONE.

[41] Kaipio K., Chen P., Roering P., Huhtinen K., Mikkonen P., Östling P., Lehtinen L., Mansuri N., Korpela T., Potdar S. (2019). ALDH1A1-related stemness in high-grade serous ovarian cancer is a negative prognostic indicator but potentially targetable by EGFR/mTOR-PI3K/aurora kinase inhibitors. J. Pathol..

[42] Pan Z., Choi S., Ouadid-Ahidouch H., Yang J.-M., Beattie J.H., Korichneva I. (2017). Zinc transporters and dysregulated channels in cancers. Front. Biosci..

[43] Groth C., Sasamura T., Khanna M.R., Whitley M., Fortini M.E. (2013). Protein trafficking abnormalities in Drosophila tissues with impaired activity of the ZIP7 zinc transporter Catsup. Development.

[44] Farra R., Maruna M., Perrone F., Grassi M., Benedetti F., Maddaloni M., Boustani M.E., Salvo Parisi S., Rizzolio F., Forte G. (2019). Strategies for delivery of sirnas to ovarian cancer cells. Pharmaceutics.

[45] Zheng D., Feeney G.P., Handy R.D., Hogstrand C., Killeb P. (2014). Uptake epithelia behave in a cell-centric and not systems homeostatic manner in response to zinc depletion and supplementation. Metallomics.

[46] Huang C., Cui X., Sun X., Yang J., Li M. (2016). Zinc transporters are differentially expressed in human non-small cell lung cancer. Oncotarget.

[47] Cui X., Zhang Y., Yang J., Sun X., Hagan J.P., Guha S., Min Li M. (2014). ZIP4 confers resistance to zinc deficiency-induced apoptosis in pancreatic cancer. Cell Cycle.

[48] McAuliffe S.M., Morgan S.L., Wyant G.A., Tran L.T., Muto K.W., Chen Y.S., Chin K.T., Partridge J.C., Poole B.B., Cheng K.-H. (2012). Targeting Notch, a key pathway for ovarian cancer stem cells, sensitizes tumors to platinum therapy. Proc. Natl. Acad. Sci. USA.

[49] Piazzi G., Bazzoli F., Ricciardiello L. (2012). Epigenetic silencing of Notch signaling in gastrointestinal cancers. Cell Cycle.

[50] Aithal M.G., Rajeswari N. (2013). Role of Notch signalling pathway in cancer and its association with DNA methylation. J. Genet..

[51] Wakabayashi N., Chartoumpekis D.V., Kensler T.W. (2015). Crosstalk between Nrf2 and Notch signaling. Free Radic. Biol. Med..

[52] Hosseini-Alghaderi S., Baron M. (2020). Notch3 in development, health and disease. Biomolecules.

[53] Yeh C.H., Bellon M., Nicot C. (2018). FBXW7: A critical tumor suppressor of human cancers. Mol. Cancer.

[54] Wiechert A., Saygin C., Thiagarajan P.S., Rao V.S., Hale J.S., Gupta N., Hitomi M., Nagaraj B.A., DiFeo A., Justin D. (2016). Cisplatin induces stemness in ovarian cancer. Oncotarget.

[55] Saygin C., Matei D., Majeti R., Reizes O., Lathia J.D. (2019). Targeting cancer stemness in the clinic: From hype to hope. Cell Stem. Cell.

[56] Ishiguro T., Ohata H., Sato A., Yamawaki K., Enomoto T., Okamotoet K. (2017). Tumor-derived spheroids: Relevance to cancer stem cells and clinical applications. Cancer Sci..

[57] Mayer B., Klement G., Kaneko M., Man S., Jothy S., Rak J., Kerbelet R.S. (2001). Multicellular gastric cancer spheroids recapitulate growth pattern and differentiation phenotype of human gastric carcinomas. Gastroenterology.

[58] Pannuti A., Foreman K., Rizzo P., Osipo C., Golde T., Osborne B., Mieleet L. (2010). Targeting Notch to target cancer stem cells. Clin. Cancer Res..

[59] Takebe N., Miele L., Harris P.J., Jeong W., Bando H., Kahn M., Yang X.S., Percy S. (2015). Targeting Notch, Hedgehog, and Wnt pathways in cancer stem cells: Clinical update. Nat. Rev. Clin. Oncol..

[60] Takebe N., Nguyen D., Yang S.X. (2014). Targeting notch signaling pathway in cancer: Clinical development advances and challenges. Pharmacol. Ther..

[61] Agliano A., Calvo A., Box C. (2017). The challenge of targeting cancer stem cells to halt metastasis. Semin. Cancer Biol..

[62] Dwivedi A.R., Thakur A., Kumar V., Skvortsova I., Kumar V. (2020). Targeting cancer stem cells pathways for the effective treatment of cancer. Curr. Drug Targets.

[63] Jung J.G., Stoeck A., Guan B., Wu R.-C., Zhu H., Blackshaw S., Shih I.-M., Wang T.-W. (2014). Notch3 interactome analysis identified WWP2 as a negative regulator of Notch3 signaling in ovarian cancer. PLoS Genet..

[64] Shah M.M., Zerlin M., Li B.L., Herzog T.J., Kitajewski J.K., Wrightet J.D. (2013). The role of Notch and gamma-secretase inhibition in an ovarian cancer model. Anticancer Res..

[65] Groeneweg J.W., DiGloria C.M., Yuan J., Richardson W.S., Growdon W.B., Sathyanarayanan S., Foster R., Rueda B.R. (2014). Inhibition of notch signaling in combination with Paclitaxel reduces platinum-resistant ovarian tumor growth. Front. Oncol..

[66] Anglesio M.S., Wiegand K.C., Melnyk N., Chow C., Salamanca C., Prentice L.M., Senz J., Yang W., Spillman M.A., Cochrane D.R. (2013). Type-specific cell line models for type-specific ovarian cancer research. PLoS ONE.

[67] Mitra A.K., Davis D.A., Tomar S., Roy L., Gurler H., Xie J., Lantvit D.D., Cardenas H., Fang F., Liu Y. (2015). In vivo tumor growth of high-grade serous ovarian cancer cell lines. Gynecol. Oncol..

[68] Bae Y., Yang T., Zeng H.-C., Campeau P.M., Chen Y., Bertin T., Dawson B.C., Munivez E., Tao J., Lee B.H. (2012). miRNA-34c regulates Notch signaling during bone development. Hum. Mol. Genet..

[69] Chen S., Lee B.H., Bae Y. (2014). Notch signaling in skeletal stem cells. Calcif. Tissue Int..

[70] Swiatek P.J., Lindsell C.E., del Amo F.F., Weinmaster G., Gridley T. (1994). Notch1 is essential for postimplantation development in mice. Genes Dev..

[71] Hamada Y., Kadokawa Y., Okabe M., Ikawa M., Coleman J.R., Tsujimoto Y. (1999). Mutation in ankyrin repeats of the mouse Notch2 gene induces early embryonic lethality. Development.

[72] Kitamoto T., Takahashi K., Takimoto H., Tomizuka K., Hayasaka M., Tabira T., Hanaokaet K. (2005). Functional redundancy of the Notch gene family during mouse embryogenesis: Analysis of Notch gene expression in Notch3-deficient mice. Biochem. Biophys. Res. Commun..

[73] Ran Y., Hossain F., Pannuti A., Lessard C.B., Ladd G.L., Jung J.I., Minter L.M., Osborne B.A., Miele L., Golde T.E. (2017). Gamma-Secretase inhibitors in cancer clinical trials are pharmacologically and functionally distinct. EMBO Mol. Med..

[74] De Strooper B., Gutierrez L.C. (2015). Learning by failing: Ideas and concepts to tackle gamma-secretases in Alzheimer’s disease and beyond. Annu. Rev. Pharmacol. Toxicol..

[75] Villalobos V.M., Hall F., Jimeno A., Gore L., Kern K., Cesari R., Huang B., Schowinsky J.T., Blatchford P.J., Hoffner B. (2018). Long-term follow-up of desmoid fibromatosis treated with PF-03084014, an oral gamma secretase inhibitor. Ann. Surg. Oncol..

[76] Ileana Dumbrava E.E., Mills G.B., Yap T.A. (2018). Targeting gamma secretase: Has progress moved up a Notch?. Ann. Oncol..

[77] Pine S.R. (2018). Rethinking gamma-secretase inhibitors for treatment of non-small-cell lung cancer: Is notch the target?. Clin. Cancer Res..

[78] Zhang Y., Bharadwaj U., Logsdon C.D., Chen C., Yao Q., Min Li M. (2010). ZIP4 regulates pancreatic cancer cell growth by activating IL-6/STAT3 pathway through zinc finger transcription factor CREB. Clin. Cancer Res..

[79] Cai H., Xu Y. (2013). The role of LPA and YAP signaling in long-term migration of human ovarian cancer cells. Cell Commun. Signal..

