# Supplementary material for: ZIP4 Is a Novel Cancer Stem Cell Marker in High-Grade Serous Ovarian Cancer
Source: Cancers (Basel). 2020 Dec 9;12(12):3692. doi: 10.3390/cancers12123692 (PMC7764492; doi:10.3390/cancers12123692)
Supplement: Supplementary file 1 [file cancers-12-03692-s001.pdf]

cancers

Supplementary Materials: ZIP4 Is a Novel Cancer Stem Cell Marker in High-Grade Serous Ovarian Cancer

Qipeng Fan, Wen Zhang, Robert E. Emerson and Yan Xu

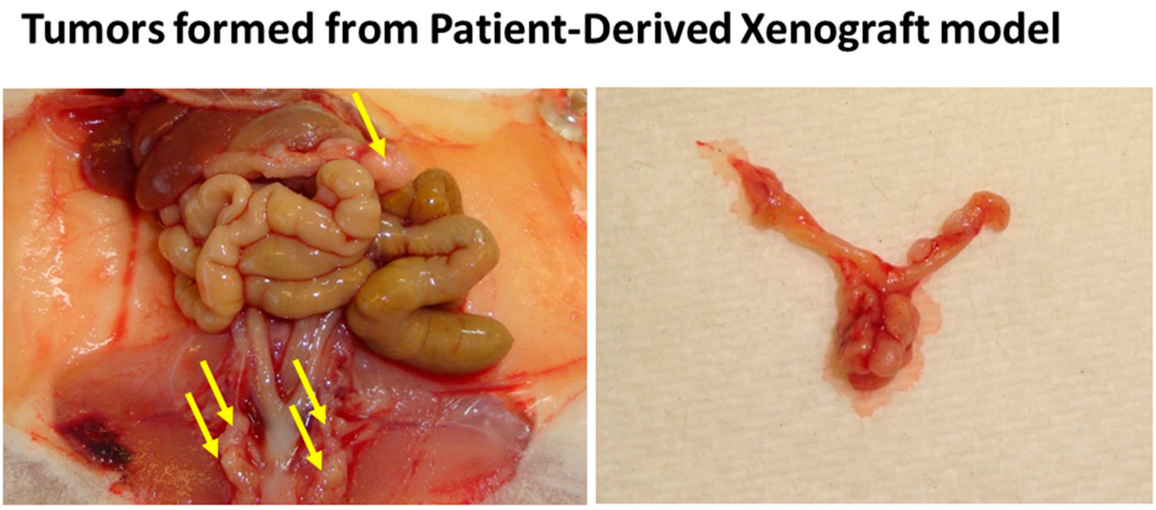

Figure S1. Representative photographs of tumors formed in Patient-Derived Xenograft model.

Figure 1

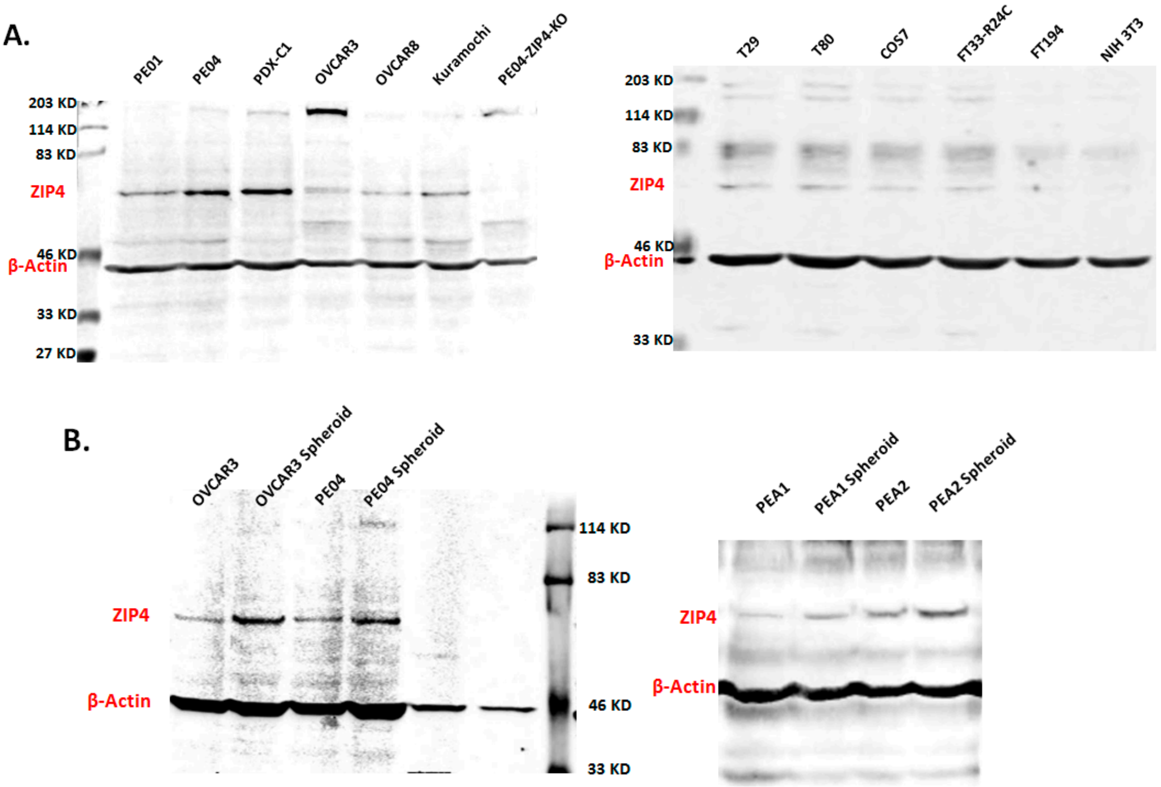

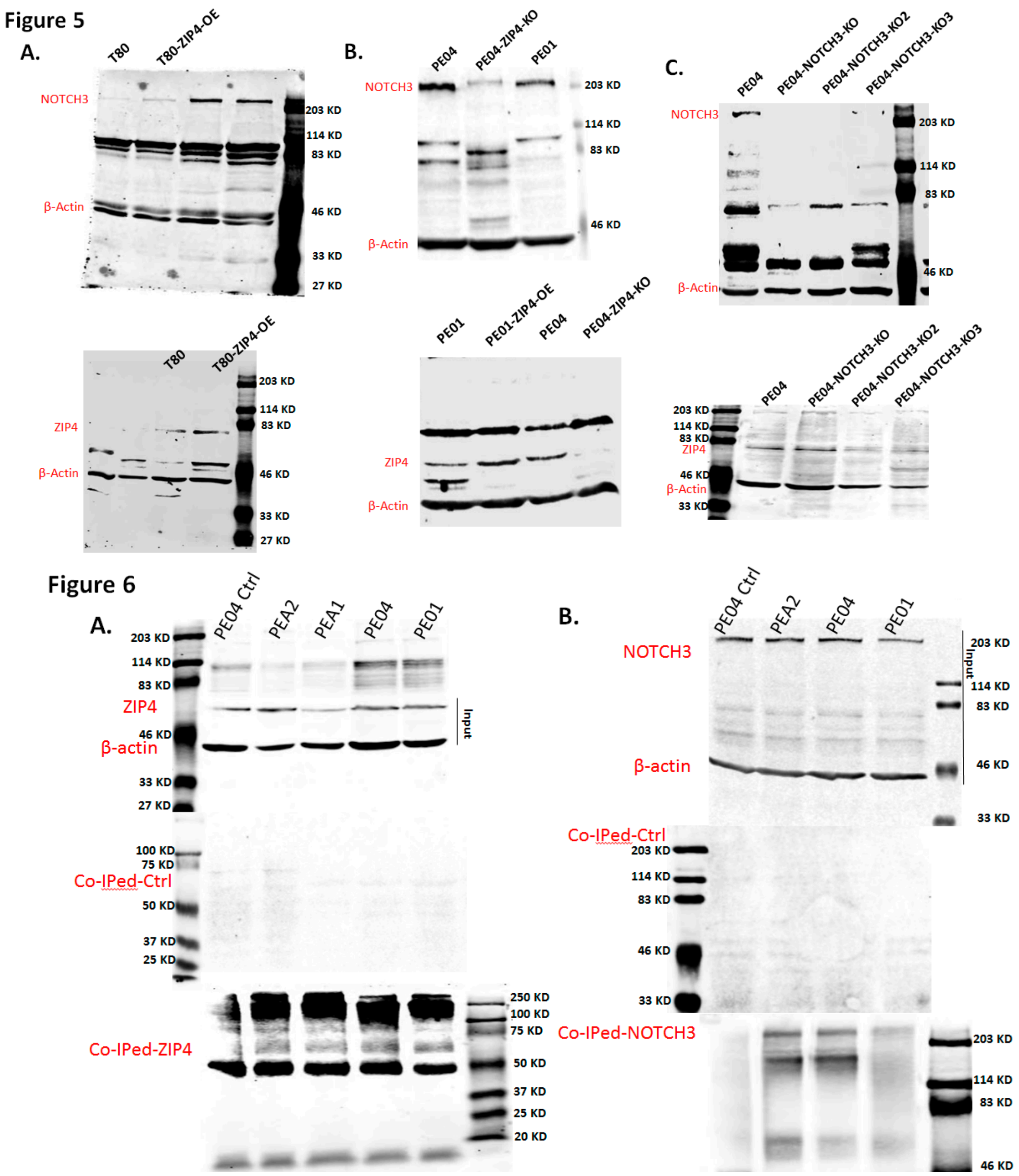

Figure S2. Uncropped Western blots. The Western blots showing all the bands with molecular weight markers for Figs 1, 5, and 6.

Publisher's Note: MDPI stays neutral with regard to jurisdictional claims in published maps and institutional affiliations.

© 2020 by the authors. Licensee MDPI, Basel, Switzerland. This article is an open access article distributed under the terms and conditions of the Creative Commons Attribution (CC BY) license (http://creativecommons.org/licenses/by/4.0/).
